# Genome-Wide Discovery of Drug-Dependent Human Liver Regulatory Elements

**DOI:** 10.1371/journal.pgen.1004648

**Published:** 2014-10-02

**Authors:** Robin P. Smith, Walter L. Eckalbar, Kari M. Morrissey, Marcelo R. Luizon, Thomas J. Hoffmann, Xuefeng Sun, Stacy L. Jones, Shelley Force Aldred, Anuradha Ramamoorthy, Zeruesenay Desta, Yunlong Liu, Todd C. Skaar, Nathan D. Trinklein, Kathleen M. Giacomini, Nadav Ahituv

**Affiliations:** 1 Department of Bioengineering and Therapeutic Sciences, University of California, San Francisco, San Francisco, California, United States of America; 2 Institute for Human Genetics, University of California, San Francisco, San Francisco, California, United States of America; 3 Department of Epidemiology and Biostatistics, University of California, San Francisco, San Francisco, California, United States of America; 4 SwitchGear Genomics, Menlo Park, California, United States of America; 5 Department of Medicine, Division of Clinical Pharmacology, Indiana University School of Medicine, Indianapolis, Indiana, United States of America; 6 Department of Medical and Molecular Genetics, Indiana University School of Medicine, Indianapolis, Indiana, United States of America; University of Chicago, United States of America

## Abstract

Inter-individual variation in gene regulatory elements is hypothesized to play a causative role in adverse drug reactions and reduced drug activity. However, relatively little is known about the location and function of drug-dependent elements. To uncover drug-associated elements in a genome-wide manner, we performed RNA-seq and ChIP-seq using antibodies against the pregnane X receptor (PXR) and three active regulatory marks (p300, H3K4me1, H3K27ac) on primary human hepatocytes treated with rifampin or vehicle control. Rifampin and PXR were chosen since they are part of the CYP3A4 pathway, which is known to account for the metabolism of more than 50% of all prescribed drugs. We selected 227 proximal promoters for genes with rifampin-dependent expression or nearby PXR/p300 occupancy sites and assayed their ability to induce luciferase in rifampin-treated HepG2 cells, finding only 10 (4.4%) that exhibited drug-dependent activity. As this result suggested a role for distal enhancer modules, we searched more broadly to identify 1,297 genomic regions bearing a conditional PXR occupancy as well as all three active regulatory marks. These regions are enriched near genes that function in the metabolism of xenobiotics, specifically members of the cytochrome P450 family. We performed enhancer assays in rifampin-treated HepG2 cells for 42 of these sequences as well as 7 sequences that overlap linkage-disequilibrium blocks defined by lead SNPs from pharmacogenomic GWAS studies, revealing 15/42 and 4/7 to be functional enhancers, respectively. A common African haplotype in one of these enhancers in the *GSTA* locus was found to exhibit potential rifampin hypersensitivity. Combined, our results further suggest that enhancers are the predominant targets of rifampin-induced PXR activation, provide a genome-wide catalog of PXR targets and serve as a model for the identification of drug-responsive regulatory elements.

## Author Summary

Drug response varies between individuals and can be caused by genetic factors. Nucleotide variation in gene regulatory elements can have a significant effect on drug response, but due to the difficulty in identifying these elements, they remain understudied. Here, we used various genomic assays to analyze human liver cells treated with or without the antibiotic rifampin and identified drug-induced regulatory elements genome-wide. The testing of numerous active promoters in human liver cells showed only a few to be induced by rifampin treatment. A similar analysis of enhancers found several of them to be induced by the drug. Nucleotide variants in one of these enhancers were found to alter its activity. Combined, this work identifies numerous novel gene regulatory elements that can be activated due to drug response and thus provides candidate sequences in the human genome where nucleotide variation can lead to differences in drug response. It also provides a universally applicable method to detect these elements for other drugs.

## Introduction

Adverse reactions to drug treatment constitute a substantial health problem that is a leading cause of morbidity and mortality in hospitalized patients [1]. Differential expression of drug metabolizing enzymes and drug transporters is a major determinant of inter-individual drug response variability [2]–[5]. By sequestering and metabolizing drug compounds in the liver and intestine, these enzymes and transporters effectively determine whether target organs and tissues are exposed to optimal drug dosages. Several coding mutations in these proteins have been detected which lead to adverse outcomes [6]–[10] and reduced drug activity [11], [12]. Regulatory elements, including promoters and enhancers, also likely play an important role that has so far been largely uncharacterized [13], [14]. The systematic identification of drug-responsive regulatory elements would thus provide a unique resource to discover novel genetic variants that lead to differences in drug response.

The vast majority of pharmaceutical compounds are metabolized by the cytochrome P450 family (CYP) of enzymes. Of these, CYP3A4 is the most abundantly expressed in sites of drug disposition in the liver [15] and is also thought to be responsible for the metabolism of at least 50% of prescribed pharmaceuticals [16]. CYP3A4 activity can vary 5–20 fold between individuals [17] and its mRNA expression can vary as much as 120 fold [18]. Only a few single nucleotide polymorphisms (SNPs) in the immediate CYP3A4 locus have been found to be associated with CYP3A4 hepatic expression [19]–[21], suggesting that its variable expression could be caused by other genes and distant regulatory elements.

CYP3A4 is one of many targets of the nuclear receptor PXR (coded by *NR1I2*), which is expressed predominantly in the liver and intestine [22] and is essential for activating Phase I and II enzymes in response to xenobiotics. PXR's broad substrate specificity allows it to be activated by a wide variety of drugs including the antibiotic rifampin, the malaria resistance drug artemisinin, the hypolipidemic agent mevastatin, and the chemotherapeutic agent paclitaxel [2]. Relatively little is known about the mechanism by which PXR drives CYP3A4 transcription *in vivo*, although PXR response elements have been identified in the putative CYP3A4 promoter [23] and upstream *cis*-regulatory elements [24], [25] that drive its expression *in vitro*. Additional PXR responsive enhancers have been found for other CYPs [26], [27]. Chromatin immunoprecipitation followed by sequencing (ChIP-seq) of PXR-bound DNA elements in livers from mice treated with PCN (a mouse PXR agonist) identified >3,000 drug-induced binding sites [28]. ChIP-seq for other drug-associated transcription factors such as LXR, RXR and PPARA has also been carried out in mouse liver [29]. However, the inherent drug metabolism differences between mouse and humans, in particular for PXR and the mouse homolog of CYP3A4 [22], [30], [31], hinder the ability to directly translate these results to humans.

To identify PXR-associated regulatory elements in a genome-wide manner, we carried out RNA sequencing (RNA-seq) and ChIP-seq with antibodies for PXR and three different enhancer marks on primary human hepatocytes treated with rifampin or vehicle control. These included the E1A binding protein p300 (EP300/p300) which has been used to identify functional enhancers *in vivo* with high success rates [32], and two histone marks, H3K4me1 and H3K27ac. H3K4me1 marks both poised and active regulatory regions [33] while H3K27ac was shown to selectively mark active regions [34], [35]. We identified thousands of sequences that had rifampin induced ChIP-seq peaks. A reporter validation screen of proximal promoters associated with these peaks yielded only a few functional rifampin-dependent sequences. A similar assay for distal enhancers resulted in the identification of several novel drug-dependent enhancers. Analyses of nucleotide variants in selected sequences found a common African haplotype in the *GSTA* locus to possibly affect rifampin sensitivity.

## Results

We acquired induction-qualified cryopreserved human hepatocytes originating from a single male donor (see Methods). To verify their inducibility, we treated cultured cells with 10 µM rifampin or DMSO control for 24 hours and then carried out quantitative PCR (qPCR) for 84 genes involved in Phase I metabolism (Table S1). Rifampin was used to activate PXR because of the wide body of literature on this interaction [36]. Furthermore, the FDA recommends using rifampin for *in vitro* and *in vivo* enzyme and transporter induction studies during drug development (http://www.fda.gov/downloads/Drugs/GuidanceComplianceRegulatoryInformation/Guidances/ucm292362.pdf). Ten genes were induced greater than two-fold (*CYP3A4*, *CYP3A43*, *CYP3A7*, *CYP2C8*, *CYP2A13*, *CYP2C9*, *CYP24A1*, *CYP1A1*, *CYP2B6*, *CYP3A5*) and only one was downregulated (*CYP26B1*). *CYP3A4* was by far the most differentially expressed gene with a >1,000 fold induction (>10 PCR cycles difference), followed by the other members of the CYP3A family (Table S1). These results indicated that this lot of cryopreserved human hepatocytes was highly inducible by rifampin, making it suitable for subsequent RNA-seq and ChIP-seq assays.

**Figure 1:**
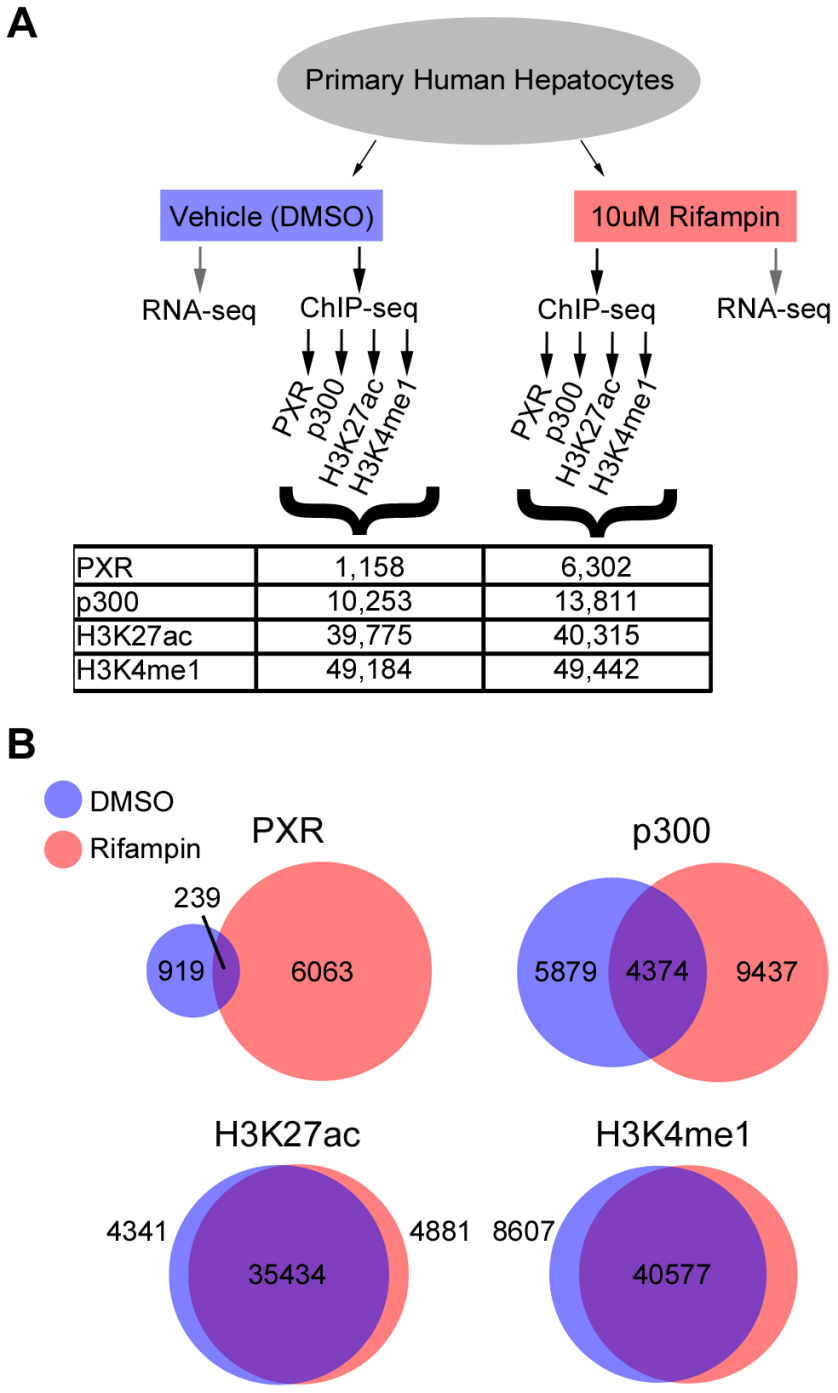
ChIP-seq summary. (A) Schematic of our RNA-seq and ChIP-seq assays, showing the various antibodies used for ChIP-seq and the total number of peaks obtained for each antibody and treatment. (B) Overlap between DMSO and rifampin treated ChIP-seq peaks for each antibody.

To identify PXR-associated regulatory elements in a genome-wide manner, we performed ChIP-seq on rifampin and DMSO-treated hepatocytes using antibodies for PXR and three enhancer marks (p300, H3K27ac, H3K4me1) (Figure 1A). The resulting libraries from these samples were sequenced along with a chromatin input sample that was used as a reference for peak calling. In addition, we collected total RNA for each treatment and carried out RNA-seq to acquire a broader view of rifampin-induced gene expression changes. To increase our reproducibility, we combined our RNA-seq results with a previously reported RNA-seq dataset for rifampin treated and non-treated human hepatocytes from seven different individuals [37] (see methods).

### Drug-associated genes are differentially expressed upon rifampin treatment

Our RNA-seq analyses found several differentially expressed genes, the majority of which are known to be involved in drug response. The number of differentially expressed genes using a p-value cutoff, after adjustment for multiple testing less than or equal to 0.05, was 157 (Table S1). Amongst them, 11 were CYPs, with the top differentially expressed gene being *CYP3A4*, similar to our qPCR results. Of the eleven differentially expressed genes identified by qPCR, seven (64%) were also found to be differentially expressed by RNA-seq. It is worth noting that two (*CYP2C9*, *CYP2C19*) of the four genes that didn't replicate in the RNA-seq data, showed a non-statistically significant induction by rifampin in our RNA-seq.

### Rifampin drives PXR and p300 recruitment at putative regulatory elements

We observed a massive recruitment of PXR binding across the genome following rifampin treatment. PXR-bound DNA fragments clustered into 1,158 discrete peaks with DMSO treated cells versus 6,302 after treatment with rifampin (Figure 1A, Table S2), with only 239 overlapping in both datasets (Figure 1B). Rifampin treatment led to a small increase in the percentage of promoters (25.6% versus 24.1%) bound by PXR and a larger increase for intronic (35.18% versus 29.9%) and exonic (5.3% versus 1.8%) regions (Table S2). In contrast, there was a reduction in the percentage of rifampin-induced PXR binding sites in intergenic regions (33.9% versus 44.2%). An analysis of the location of rifampin-induced PXR peaks found them to be enriched at transcription start sites (TSSs), but not at a particular location upstream or downstream to the TSS(Figure S1).

The binding of p300 was also more extensive after rifampin treatment, with 13,811 peaks compared to 10,253 in DMSO-treated cells (Figure 1A, Table S2). There was a larger overlap between rifampin and DMSO treated ChIP-seq peaks compared to PXR, with 4,374 (31.7%) in common between the two sets (Figure 1B, Table S2). We also observed a change in the functional distribution of binding sites, with rifampin increasing the percentage of intronic (42.6% versus 36.3%) and intergenic (38.0% versus 31.7%) p300 binding versus a small change in exons (3.8% versus 2.6%) and a reduction in promoter binding (15.5% versus 29.4%) (Table S2). This result was consistent with our observation that only 1,076 rifampin-induced p300 peaks overlapped rifampin-induced PXR peaks (Table S2).

In contrast to PXR and p300, the distribution of histone marks was relatively stable, with about 49,000 enriched islands of H3K4me1 activity and about 40,000 H3K27ac islands in both treatments (Figure 1A). We also observed a large overlap between rifampin and DMSO treated H3K4me1 (82.07%) and H3K27ac (87.89%) enriched islands (Figure 1B, Table S2). Combined, these results suggest that histone marks are more stable in response to rifampin treatment compared to PXR and p300.

We next looked at overlaps between the different ChIP-seq peaks. Amongst the 6,302 PXR rifampin treated peaks 1,037 (16%) overlapped p300 and around half overlapped histone marks (3,553 for H3K27ac and 2,942 for H3K4me1). This was similar for PXR peaks in the DMSO treated cells (Table S2). For p300 we observed a greater overlap with histone marks, with ∼70% of the peaks overlapping either H3K27ac (9,487/13,811) and H3K4me1 (9,840/13,811). In the DMSO treated cells, we observed a much higher overlap for p300 peaks with the active H3K27ac mark (9,487/10,253; 92%) versus H3K4me1 (7,789/10,253; 76%), suggesting that the p300 peaks in this condition tend to be in active regions.

### Functional validation of putative rifampin induced promoters

**Figure 2:**
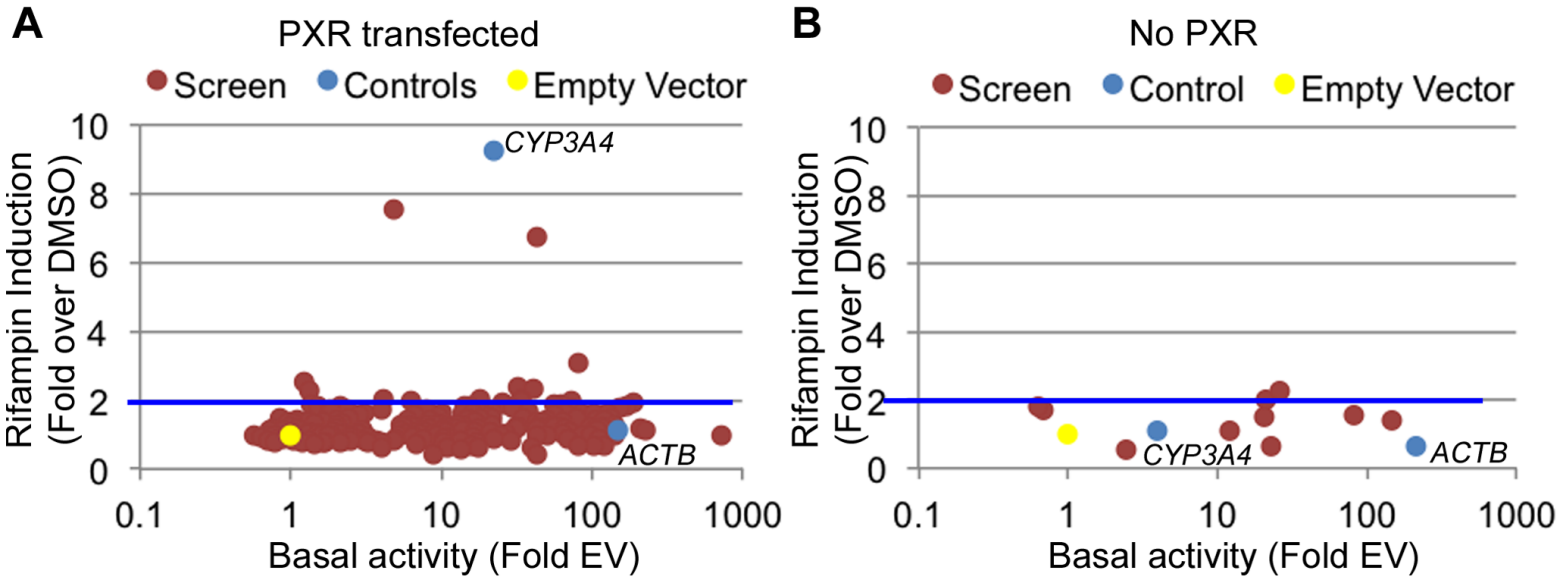
Functional promoter assays. (A) From the 227 tested promoters (red dots), 179 were found to be functional promoters (>2 fold luciferase activity above empty vector) in the DMSO treated cells (basal activity). Among those, 10 showed a >2-fold increase upon rifampin treatment. (B) The 10 promoters exhibiting rifampin induction in A were tested again without PXR. Blue dots are the positive controls (*ACTB* and *CYP3A4* promoters) and the yellow dot is the empty vector.

There are multiple examples of promoter nucleotide variants that are associated with inter-individual drug response [4], [5], [13], [38], [39]. We thus sought to identify drug-dependent promoters in our dataset which may harbor common variants with novel effects on drug response. We selected 227 promoters for 200 genes (some genes had more than one promoter; Table S3) from the LightSwitch Promoter Collection (SwitchGear Genomics) for genes whose expression was induced by rifampin or reside near rifampin-induced ChIP-seq peaks (Table S3). Of the 227 promoters, 154 overlap a PXR peak, 45 overlap p300, 164 overlap H3K27ac and 84 overlap H3K4me1 (Table S3). This library consists of ∼1,000 bp proximal promoter fragments cloned into pLightSwich_Prom vector (see Methods, Table S3). We also included two positive controls: 1) The beta-actin promoter (*ACTB*), a strong constitutive promoter that should not be induced by rifampin and 2) The *CYP3A4* proximal promoter, which is known to be induced by rifampin. We tested the 227 promoters in HepG2 cell lines co-transfected with human PXR and treated with rifampin or vehicle control (DMSO). Out of the 227 tested promoters, 179 were found to be functional promoters (>2 fold luciferase activity above empty vector) in the DMSO treated cells (Figure 2A, Table S3). Among those promoters, only 10 exhibited >2 fold increase in promoter activity upon rifampin treatment including our *CYP3A4* proximal promoter control (Figure 2A). To confirm that the effects of rifampin were mediated through PXR, we also tested the 10 rifampin-induced promoters (including *CYP3A4*) in a similar assay, only this time without co-transfecting human PXR, and found only 2 of them to be induced by rifampin (>2 fold increase in promoter activity upon rifampin treatment) and at much lower levels (Figure 2B, Table S3). In addition, the *CYP3A4* promoter was also not induced by rifampin in this assay. In both experiments, our *ACTB* control was a strong promoter, but not induced by rifampin. The overall lack of rifampin-sensitive promoters and previous results finding a role for enhancers in driving this drug response [24]–[27] suggests that other regulatory sequences, such as enhancers, may be involved in driving the effects of rifampin treatment on gene expression.

### Rifampin induced regions reside near drug-associated genes

**Figure 3:**
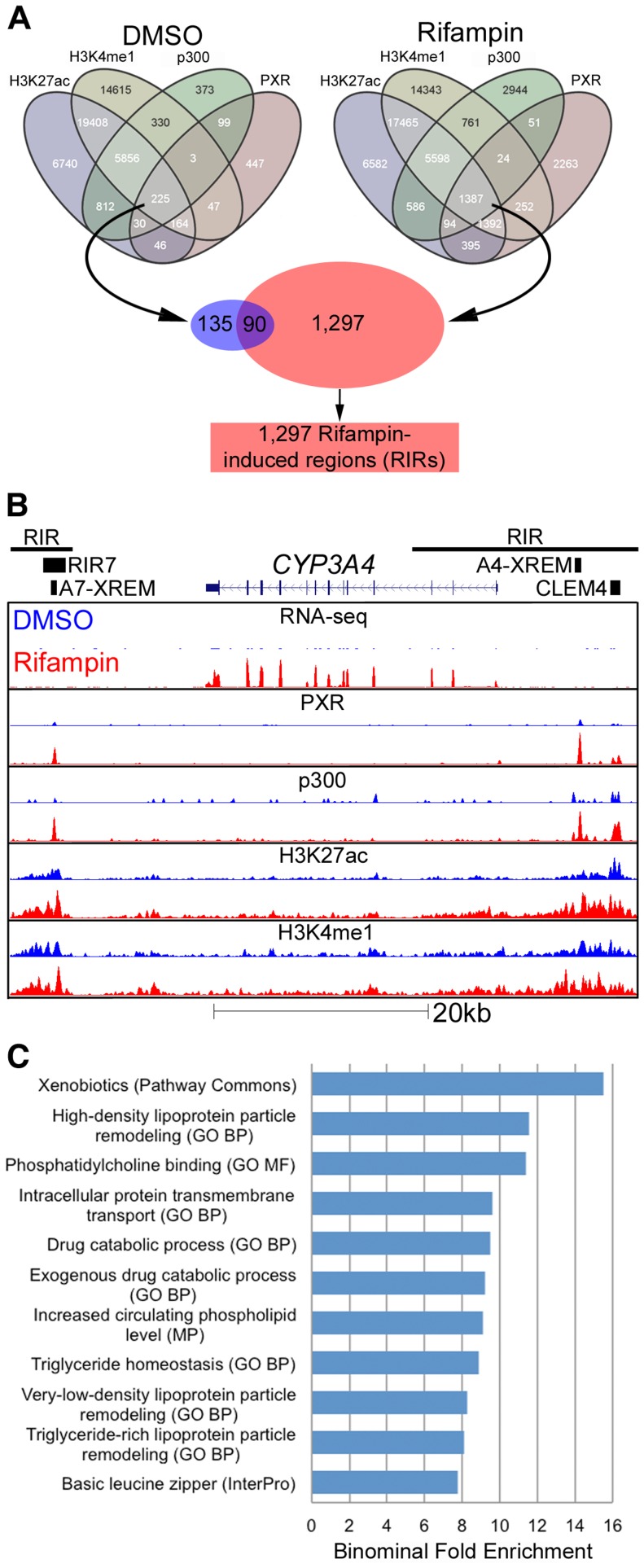
Genomic characterization of RIRs. (A) Overlap between the four different ChIP-seq marks for DMSO and rifampin treated human hepatocytes. Sequences having all four marks in the rifampin treated datasets, were termed rifampin induced regions (RIRs). (B) The *CYP3A4* locus shows increased *CYP3A4* expression in the rifampin treated hepatocytes (red) versus DMSO treated cells (blue) as observed through RNA-seq, as well as ChIP-based enrichment of PXR, p300, H3K27ac and H3K4me1 in rifampin-(red) versus DMSO-treated cells (blue). The xenobiotic responsive enhancer module (XREM) [24], the −11 kb constitutive liver enhancer module of *CYP3A4* (CLEM4) [25] and the *CYP3A7* XREM [26] are also shown. RIRs are depicted as black lines and the cloned RIR7 fragment as a black rectangle. (C) An analysis of RIRs using GREAT [40] identified enriched biological processes associated with drug response. Shown are only terms that have binomial fold enrichment >6 and FDR adjusted P-values<5×10^−3^. A full listing can be seen in Table S4.

Since most of the promoters tested in our assay did not demonstrate increased activity in the presence of rifampin, we broadened our search for inducible regulatory elements to include enhancers. To be more stringent in our analyses, we selected regions across the genome which showed PXR rifampin-induced binding in addition to all three enhancer marks. For both the DMSO and rifampin treatments, we generated a merged track of all four marks, with each region in the track overlapping one to four peaks/island. If, for example, a p300 peak is near a PXR peak, but they don't overlap, while both overlap a H3K4me1 and/or a H3K27ac island, they were considered all as one region. Only 225 such regions were present in the DMSO treatment, while 1,387 were identified in the cells treated with rifampin (Figure 3A). Of the latter group, 1,297 regions were exclusive to the rifampin treatment and termed Rifampin-Induced Regions (RIRs) for downstream analyses.

*CYP3A4* is by far the most well studied target of PXR, with well characterized regulatory sequences: the proximal promoter, a −7.5 kb upstream xenobiotic responsive enhancer module (XREM) [24], and a −11 kb constitutive liver enhancer module of *CYP3A4* (CLEM4) [25]. A second potential XREM, putatively regulating *CYP3A7*, was additionally identified intergenically between *CYP3A7 and CYP3A4* [26]. Our ChIP-seq data completely recapitulates this picture of regulation in primary human hepatocytes, with two large RIRs encompassing multiple rifampin-induced peaks (Figure 3B). It is also worth noting that the *CYP3A4* locus is one of the few in which we observed a substantial difference in rifampin-induced enrichment of the H3K4me1 and H3K27ac marks.

To identify enriched biological pathways and functions within the set of 1,297 RIRs, we carried out a genomic analysis using the Genomic Regions Enrichment of Annotations Tool (GREAT [40]). Our top enriched term (p-value 1.85×10^−9^; binominal fold enrichment), originating from Pathway Commons (http://www.pathwaycommons.org), was ‘xenobiotics’ (Figure 3C). This was attributed to RIRs residing near the following genes: *ABCB4*, *ACSL1*, *ADH1A*, *ADH6*, *AKR1C2*, *AKR1C3*, *ALDH1A1*, *CNDP2*, *CYP26A1*, *CYP2A6*, *CYP2B6*, *CYP2C19*, *CYP2C8*, *CYP2C9*, *CYP2W1*, *CYP3A4*, *CYP3A7*, *CYP4F12*, *CYP4F3*, *CYP7A1*, *GCLC*, *GCLM*, *GSTA2*, *GSTO1*, *GSTO2*, *HNF4A*, *MAT1A*, *MAT2A*, *MGST2*, *MGST3*, *NCEH1*, *NNMT*, *PAPSS2*, *PTGIS*, *SLC35D1*, *SULT1B1*, *SULT2A1*, *UGDH*, *UGT1A1*. In addition, we observed significant (FDR adjusted p-value≤0.05) gene ontology enrichment terms for drug catabolic processes and other terms fitting with drug response (Figure 3C, Table S4). We performed a similar analysis for RIR neighboring genes using QIAGEN's Ingenuity Pathway Analysis (IPA, QIAGEN Redwood City, www.qiagen.com/ingenuity) and found enrichment for the PXR/RXR Activation Canonical pathway (Figure S2, Table S5). Combined, these results suggest that our RIRs are enriched near drug-associated genes.

### Functional validation of putative rifampin induced enhancers

Although ChIP-seq is a valuable tool for the identification of putative regulatory elements, functional studies are essential for the validation of such sequences. We selected forty-nine putative enhancer sequences for validation using two different selection criteria: 1) Forty-two RIRs residing near drug-associated genes as manually determined by the literature. 2) Seven rifampin-induced PXR ChIP-seq peaks harboring SNPs that are in linkage disequilibrium (LD) with pharmacogenomics or drug related genome-wide association study (GWAS) lead SNPs, termed GWAS linked peaks (GLPs) (Table S6). Previous studies have shown that several GWAS SNPs reside near potential regulatory elements that encompass SNPs that are in LD with the lead GWAS SNP [41]–[43]. To increase our chances to identify these regulatory elements, we used our rifampin treated PXR-ChIP-seq dataset, instead of the RIRs, since it had a larger number of peaks. SNPs in LD with pharmacogenomics GWAS hits were significantly enriched near rifampin-dependent PXR peaks compared to SNPs in LD with non-pharmacogenomic GWAS hits (p<0.0001, Chi-squared test). None of the sequences chosen overlapped promoter regions (i.e. were within −2500/+500 bp of a TSS).

Candidate enhancer sequences were cloned into the pGL4.23 (Promega) enhancer assay vector, which contains a minimal promoter followed by the luciferase reporter gene. Since the peaks (or islands) enriched for the two histone marks are relatively long (average length ∼5 kb, Table S2), we selected shorter sequences within each RIR that encompass only the PXR/p300 peaks along with additional flanking sequence, up to 500 bp on each side of the peak. We also used different positive controls: 1) The *ApoE* liver enhancer [44] whose activity should not be enhanced by rifampin. 2) A *CYP3A4* promoter-enhancer combination (p3A4-362(7836/7208ins) [24] (Table S7) whose activity should be increased by rifampin treatment. All constructs were tested for their enhancer activity in HepG2 cells transfected with human PXR and treated with either rifampin or DMSO, as previously done for the promoter screen.

**Figure 4:**
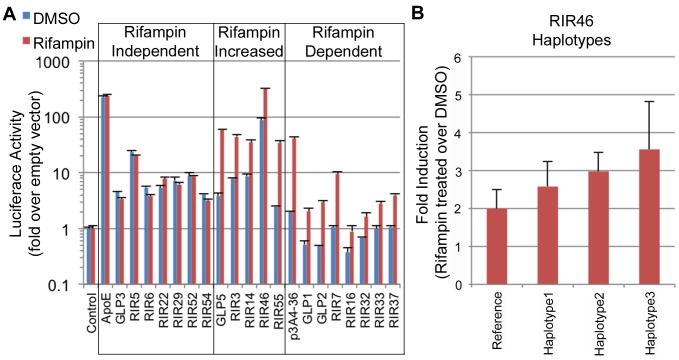
Functional enhancer assays. (A) RIRs or GLPs that showed positive enhancer activity in our assays. These were divided into three classes: 1) rifampin independent, where the addition of rifampin did not change enhancer activity; 2) rifampin increased, where rifampin increased enhancer activity; 3) rifampin dependent, when only upon rifampin treatment did the sequence show significant enhancer activity. An empty vector was used as a negative control and the *ApoE* liver enhancer [44] and a *CYP3A4* promoter-enhancer combination (p3A4-362(7836/7208ins) [24] labeled as p3A4-36, were used as positive controls. (B) Haplotype 3 in RIR46 shows increased enhancer activity (adjusted p-value (FDR) = 0.095 from post test when compared to the reference haplotype; one-way ANOVA).

Out of the 49 sequences tested, 19 (38.7%) showed significant reporter expression levels versus the empty vector (≥2 two fold) in either condition: 15 RIRs and 4 GLPs (Figure 4A, Table S7). Among these 19 positive enhancers, we observed three types of enhancers: 1) Seven enhancers that were active at similar levels with DMSO and rifampin, termed ‘rifampin independent’. 2) Five enhancers that were active without rifampin, but whose expression levels significantly increased upon rifampin treatment, termed ‘rifampin increased’. 3) Seven enhancers that were active only when treated with rifampin and were called ‘rifampin dependent’. Two of these (GLP1 and GLP2) are located in the *CYP2C* locus (Figure S3) and contain SNPs in LD with pharmacogenomics GWAS SNPs for warfarin maintenance dose [45], [46], acenocoumarol maintenance dose [47] and response to clopidogrel therapy [48]. Combined, these results show that enhancer activity can be modulated by rifampin.

### Genetic variants in enhancers drive differential expression of PXR target genes and may explain variability in drug response

We next determined whether common nucleotide variation within functional, drug-dependent enhancers could alter their activity. For these experiments we selected five enhancers that were either rifampin increased or dependent and near important drug-response genes. These included RIR7, which overlaps the putative *CYP3A7* XREM (Figure 3B; chr7: 99339411–99341549; hg19) [26] and was rifampin dependent in our assays. We also selected RIR46, which is located in the glutathione S-transferase alpha (*GSTA*) locus near *GSTA2* (chr6: 52609942–52611507; hg19) (Figure S4) and was rifampin-increased in our assays. The GSTA family of enzymes are known to be involved in the metabolism of various xenobiotics [49]. We also selected three different GLP sequences: GLP1, 2, and 5. Both GLP1 (chr10:96507473–96508107; hg19) and GLP2 (chr10:96696182–96696970; hg19) are located in the *CYP2C* locus (Figure S3), which harbors several CYP metabolizing enzymes and has been analyzed extensively in various pharmacogenomic studies. GLP5 (chr2:234672744–234673398; hg19) harbors a single SNP, rs3771341, that is in LD with several GWAS lead SNPs correlated with altered bilirubin levels [50]–[53] and was rifampin-increased in our study. This element is located in the UDP glucuronosyltransferase 1 family, polypeptide A cluster (UGT1A), ∼4 kb upstream of the *UGT1A1* transcription start site (Figure S5). UGT1A enzymes have important roles in the metabolism of xenobiotics and both coding and promoter variants within them have been associated with adverse drug reactions [54].

We determined common haplotypes in all five sequences using the phased 1000 Genomes data (Table S8). Common haplotypes for all five sequences (Table S8) were then cloned into our enhancer assay vector (pGL4.23) either by amplifying DNA from individuals from various ethnic backgrounds from the studies of pharmacogenomics in ethnically diverse populations (SOPHIE) cohort [55] or by site-directed mutagenesis and sequence verified. The sequences were then tested for enhancer activity in HepG2 cells transfected with human PXR and treated with either rifampin or DMSO, and compared to the ancestral haplotype. Out of the five tested enhancers, one haplotype in RIR46 showed a substantial difference in enhancer activity. For RIR46, we observed 1.85-fold (4.01/2.16) increase in response to rifampin for haplotype 3, however after adjusting for multiple testing the variance in the response was too high to be significant (adjusted p-value = 0.095 by FDR; ANOVA; Figure 4B, Table S8). This haplotype is present at a frequency of 6.7% in the 1000 Genomes AFR population. It is worth noting that our success rate in finding haplotypes that significantly alter enhancer activity was low. Nonetheless, the observation that a haplotype in RIR46 could possibly affect enhancer activity suggests that nucleotide variants in these enhancers could lead to differential enhancer activity.

## Discussion

By carrying out RNA-seq and ChIP-seq on rifampin and DMSO treated human hepatocytes, we have uncovered numerous drug-dependent regulatory elements. We observed that promoters bearing a rifampin-dependent signature were largely unable to independently induce the expression of a reporter upon rifampin treatment. This result suggested that other gene regulatory elements, such as enhancers, could constitute the predominant group of target sequences that are activated by this drug. An analysis of nucleotide variation in these enhancers showed that specific variants could affect enhancer activity, raising the possibility that nucleotide variants in enhancers could contribute to pharmacogenomic phenotypes more broadly.

Our RNA-seq analyses that combined eight different individuals identified 157 differentially expressed genes, using a p-value cutoff that adjusted for multiple testing less than or equal to 0.05 (Table S1). Many of these genes are known to be involved in drug response and the top differentially expressed gene was *CYP3A4*, fitting with its role as the most abundantly expressed gene in sites of drug disposition in the liver [15]. The number of differentially expressed genes (157), using our 0.05 cutoff, was much lower than the number of RIRs (1,297) and PXR and p300 rifampin treated ChIP-seq peaks. This difference could be attributed to having multiple regulatory elements regulating the same gene. It could also be due to our conservative RNA-seq differential expression cutoff, which if relaxed would increase our gene list. Another cause for this can be that several of our identified peaks are not functional regulatory elements. Our functional characterization for example, found only 19 of the 49 tested sequences (38.7%) to have significant reporter expression levels versus the empty vector (≥2 two fold). Of note though, that this could also be due to the different cell types and conditions that were used in this assay, as later described.

Similar studies have been conducted to detect environmentally induced regulatory elements [28], [56]–[59]. For example, testing for changes in estrogen receptor α and RNA polymerase II occupancy due to estradiol, tamoxifen or fulvestrant treatment in MCF-7 cells, found differences in ligand regulation with tamoxifen leading to downregulation while fulvestrant increased RNAPII occupancy [58]. In our study, we observed a large rifampin-induced recruitment of PXR and p300 binding across the genome. This was particularly notable in the *CYP3A* locus, which was radically altered by rifampin treatment. For the most part, the regulatory hotspots that we identified in this region are consistent with established literature. It is worth noting, however, that we saw almost equal recruitment of PXR and p300 to the XREMs that putatively regulate *CYP3A4* and *CYP3A7*, despite the fact that we observed substantially less CYP3A7 mRNA induction by qPCR and RNA-seq.

We did not observe large changes in the assayed histone marks, H3K4me1 and H3K27ac. This could be attributed to these sites being poised to be activated by various xenobiotic responses, though H3K27ac has been previously shown to mark active enhancers [34], [35]. To get at these differences more systematically, we performed a second analysis in which H3K4me1 and H3K27ac peaks were called for the rifampin treatment using the DMSO treatment as the control (Table S9). We observed 110 differential islands for H3K27ac, and only 2 for H3K4me1 (one of which was in the *CYP3A4* locus). The fact that we observed more rifampin-induced peaks for H3K27ac is consistent with its hypothesized role in marking active enhancers. Combined, these results suggest that while these two marks are incredibly stable in the face of massive changes in PXR/p300 binding, there are still measurable drug-induced changes in chromatin structure.

Previous reports have found enhancers to be responders of drug treatment [24]–[27]. Our functional validation results broadly suggest that promoters on their own are largely incapable of driving the effects of rifampin induction. Instead, our results imply that enhancers appear to be induced by rifampin, and through their interaction with promoters, drug response genes are activated. We tested over 200 promoters of genes based on relatively loose selection criteria: that their respective genes either showed increased expression following rifampin treatment, were tagged by our rifampin ChIP-seq peaks, or were near them. On the other hand, for enhancers we required that candidates had all four marks. While it is possible that our promoter selection criteria missed out on important drug response promoters, we would still expect to achieve a higher rifampin induction success rate than what we observed in our assays (10/227; 4.4%). While our assays may have not been ideal for these purposes, i.e. testing promoters *in vitro* in PXR-transfected HepG2 cells, both our negative and positive controls showed the expected results (Figure 2A). Furthermore, rifampin-induced promoters tested in non-PXR transfected conditions showed much lower induction by the drug (Figure 2B). Further systematic assays including ones carried out *in vivo* will be needed to better address this hypothesis.

While 12 out of the 19 functional sequences identified by our screen exhibited basal enhancer activity, 7 sequences exhibited enhancer activity only upon rifampin treatment (Figure 4A). These sequences would not have been identified by conventional ChIP studies conducted in physiological conditions, nor would they be validated in functional assays without drug treatment. Together our results suggest that ChIP-seq datasets are dependent on the environmental conditions in which they were performed, and that there are likely many hidden enhancers which only become active following a specific stimulus.

We identified several functional enhancers that were rifampin-increased or rifampin-induced whose location was near pharmacogenomic-associated variants. One of these elements is RIR46, which resides near GSTA2, a Phase II enzyme involved in the detoxification of numerous drugs [49], and is thus a likely target. Coding variants in *GSTA2* have been shown to affect its detoxification efficiency [60], [61] and promoter variants in *GSTA2* have been suggested to affect its expression levels [62], [63]. We identified a haplotype present in the 1000 Genomes AFR population that confers increased rifampin sensitivity (Figure 4B). Both *GSTA2* and *GSTA1* (which is 28 kb downstream to GSTA2), have been shown to have important roles in catalyzing carcinogenic substrates and nucleotide variation in them has been shown to be associated with cancer [60], [64]. Previous work has shown a significant difference in the distribution of coding SNPs in both these genes between African Americans and Caucasians [60]. However, these coding SNPs are not associated with prostate cancer disease status [60], suggesting that other factors could be playing a role. Future studies could examine whether variants in RIR46 or other RIRs are associated with these phenotypes.

It is worth noting that there are several caveats to our study. Our ChIP-seq experiments only analyzed hepatocytes from a single donor at a single time point (24 hours post treatment), selected for being commonplace in rifampin induction studies. It is possible that there are enhancers that play a role much earlier than 24 hours post-treatment. To test regulatory sequences in reporter assays, it was also necessary to clone smaller fragments from each RIR bearing the PXR and p300 peaks. It is possible that we missed flanking sequences that were essential for enhancer function. Furthermore, because we had a limited amount of primary hepatocytes from the same donor, our reporter assays were carried out in PXR-transfected HepG2 cells, which could result in false negatives. Finally, we employed the pGL4.23 vector, which is a commonly used enhancer assay vector, but possesses a very short TATA-box containing minimal promoter that may not be compatible with all of the enhancer sequences tested.

Promoter regions upstream of drug metabolizing enzymes and transporters are presumed to be the major targets of xenobiotic activators of PXR, such as rifampin. Our work challenges this conception and strongly supports the idea that the direct targets could be enhancer elements, which may subsequently interact with promoters to enhance gene transcription. Combined, our results show that ChIP-seq in combination with drug treatment has a large potential in identifying novel regions in the genome associated with drug response. These regions can provide exceptional candidates for the detection of nucleotide variants associated with inter-individual differences in drug response. Our methodology could easily be adapted to other drugs/target/tissue combinations. With whole genome datasets becoming more widely used as a clinical toolbox, the ability to highlight these important drug response regions in the genome is of extreme importance.

## Materials and Methods

### Primary hepatocyte culture

Cryopreserved human hepatocytes from a 19 year old Caucasian male donor with no history of medications (Lot Hu8080, Life Technologies) were thawed in CHRM recovery media (Cat# CM7000, Life Technologies) and cultured in CHPM plating media (Cat#CM9000, Life Technologies) on 6-well collagen-coated plates (Life Technologies). After 6 hours, the media was swapped with maintenance media, consisting of phenol-free Williams E media containing culture incubation supplements (Cat#CM4000, Life Technologies) and 0.01% DMSO or 10 µM rifampin (Sigma) for 24 hours. The rifampin dose (10 µM for 24 hours) was used based on previously reported assays achieving high induction rates in human hepatocytes [65], [66]. Dexamethasone, which is included separately with the culture incubation supplements, was not added to the media as it can activate PXR.

### Quantitative PCR validation

Cultured hepatocytes were treated with DMSO or rifampin for 24 hours in triplicate. The cells were then washed with PBS, and lysed directly with Buffer RLT from the RNAeasy mini kit (Qiagen) with the on-column DNase digestion step. One µg of total RNA was used to generate cDNA using the RT^2^ First Strand Kit (Qiagen). Gene expression levels for 84 genes of interest was determined using the “Drug Metabolism: Phase I Enzymes” RT^2^ Profiler PCR Array (Qiagen). Five housekeeping genes (*B2M*, *HPRT1*, *RPL13A*, *GAPDH*, *ACTB*) were used to control for loading. Fold induction was calculated using the ΔΔC_t_ method [67].

### RNA-seq

Total RNA was acquired as described for qPCR for two replicates each of DMSO and rifampin treated hepatocytes. Libraries were made with ScriptSeq v2 RNA-Seq Library Preparation Kit (Epicenter). Briefly, 3–5 ug of total RNA were subjected to rRNA removal using Ribo-Zero Magnetic Kit (Epicenter) prior to library construction. 5 ng of the rRNA-depleted sample was fragmented enzymatically and annealed with random hexamer to create the first strand of cDNA. Upon removal of the RNA template transcript by RNase, Terminal-Tagging Oligo (TTO), a known 5′-sequence tag, a 3′-random sequence, and a terminally blocked 3′ end to prevent priming of DNA synthesis, was added to create cDNA with known sequence tags at their 5′ and 3′ ends for directionality. Upon purification, adaptors with barcodes were added to cDNA fragments and enriched by 15 cycles of PCR. Sequencing was carried out on an Illumina HiSeq. The resulting reads were demultiplexed and aligned to the human genome (hg19) using TopHat v2.0.10 [68]. Read counts for each gene in the RefSeq annotation were obtained using NGSUtils [69] so as to allow comparison to the RNA-seq data from the other 7 primary hepatocytes treated with and without rifampin and sequenced with SOLiD as described in [37]. Analysis for differential expression across the nine replicates was performed using DESeq2 [70]. DESeq2 was chosen due to its capability in handling multifactorial experimental designs, in this case treated with rifampin versus control and SOLiD sequences versus Illumina. DESeq2 was then used to perform a likelihood ratio test between the model of treatment plus sequencing type versus the simplified model of just sequencing type in order to identify genes differentially expressed upon treatment with rifampin.

### ChIP-seq

Twelve million cells (an entire 6 well plate) per immunoprecipitation were fixed with 1% formaldehyde for 15 min and quenched with 0.125 M glycine. The remainder of the ChIP-seq protocol was carried out by Active Motif Inc., as follows. Chromatin was isolated by adding lysis buffer, followed by disruption with a Dounce homogenizer. Lysates were sonicated and the DNA sheared to an average length of 300–500 bp. Genomic DNA (Input) was prepared by treating aliquots of chromatin with RNase, proteinase K and heat for de-crosslinking, followed by ethanol precipitation. Pellets were resuspended and the resulting DNA was quantified on a NanoDrop spectrophotometer. Extrapolation to the original chromatin volume allowed quantitation of the total chromatin yield.

An aliquot of chromatin (30 ug) was pre-cleared with protein G agarose beads (Invitrogen). Genomic DNA regions of interest were isolated using 4 ug of antibody against PXR (sc-25381, Santa Cruz), 4 ug of antibody against p300 (Cat#sc-595, Santa Cruz), 20 ug of antibody against H3K27ac (Cat#ab4729, Abcam) and 5 ug antibody against H3K4me1 (Cat#ab8895, Abcam). Due to the limited amount of cryopreserved human hepatocytes from this donor, we elected to do various enhancer-associated ChIP-seq marks instead of additional replicates. Complexes were washed, eluted from the beads with SDS buffer, and subjected to RNase and proteinase K treatment. Crosslinks were reversed by incubation overnight at 65 C, and ChIP DNA was purified by phenol-chloroform extraction and ethanol precipitation.

ChIP and input DNAs were prepared for amplification by converting overhangs into phosphorylated blunt ends and adding an adenine to the 3′ ends. Illumina adaptors were added and the library was size-selected (175–225 bp) on an agarose gel. The adaptor-ligated libraries were amplified for 18 cycles. The resulting amplified DNAs were purified, quantified, and tested by qPCR at the same specific genomic regions as the original ChIP DNA to assess quality of the amplification reactions. Amplified DNA libraries were sequenced on the Illumina Genome Analyzer II.

The 36-nt sequence reads identified by the Sequencing Service were mapped to the genome using the ELAND (Illumina). Default settings were used: only tags that map uniquely, have no more than 2 mismatches, and that pass quality control filtering were considered.

PXR and p300 peaks were called against input using MACS version 1.4 [71] and for H3K27ac and H3K4me1 using SICER version 1.1 [72]. A p-value cutoff of 1×10^−7^ was used for MACS and an FDR of 0.01% was used as a cutoff for SICER, and the remaining parameters set to the default. For the alternate analysis presented in Table S9, we used a more stringent threshold in SICER of 1×10^−10^.

For the analysis of the distribution of PXR ChIP-seq peaks following rifampin treatment, we used knownGene from the UCSC Genome Browser to define the transcription start site (TSS) as the center and pybedtools [73] for the calculations followed by matplotlib (http://matplotlib.org) to plot.

### Reporter plasmids

Selected promoters were obtained from the LightSwitch Promoter Collection (SwitchGear Genomics) and sequence verified in the pLightSwitch_Prom vector. Candidate enhancers were amplified from human genomic DNA (Roche) using oligonucleotides designed in Primer3 with 16 bp overhangs (5′-GCTCGCTAGCCTCGAG-3′ and 5′CGCCGAGGCCAGATCT-3′) complementary to the sequence flanking the BglII and XhoI sites in the pGL4.23 vector (Promega). Primers were designed to encompass the PXR/p300 ChIP-seq peak plus up to 500 bp of sequence on either side of the peak. PCR products were cleaned using the QIAquick PCR Purification Kit (Qiagen) and cloned into BglII and XhoI digested pGL4.23 using the Infusion HD cloning system (Clontech). Haplotypes were generated either by PCR amplification of DNA from various ethnic individuals or by site-directed mutagenesis using mutant primers amplified by PfuUltra High Fidelity DNA polymerase (Agilent) followed by DpnI digestion of the parental DNA (New England Biolabs) and transformation into competent cells. Sequences were verified by Sanger sequencing using RVPrimer3 (5′-CTAGCAAAATAGfGCTGTCCC-3′) and a custom reverse primer (5′-TCTTCCATGGTGGCTTTACC-3′).

### Identification of GWAS-associated PXR peaks

We manually curated the NHGRI GWAS catalog [74], dated December 15^th^, 2011, to identify 397 SNPs from 92 pharmacogenomic GWAS studies. Using data from the 1000 Genomes March 2012 Interim release (http://1000genomes.org), we identified a larger set of SNPs in linkage disequilibrium with those SNPs (r^2^>0.8) in the race/ethnicity corresponding to the pharmacogenetics study. Ten rifampin-induced PXR peaks overlapped at least one of these GWAS-LD SNPs (Table S6) and seven were selected for functional enhancer assays. To determine enrichment, we compared overlap of the LD blocks defined by the 397 lead SNPs from pharmacogenomic GWAS to 5,117 lead SNPs from non-pharmacogenomic GWAS. These assignments were curated manually. Each list of SNPs was intersected with the 6,302 rifampin-induced PXR peaks, resulting in 27 and 162 overlapping SNPs respectively. Using a two-tailed Chi-squared test with Yates' correction, we found the enrichment to be highly significant (p<0.0001).

### Luciferase reporter assays

HepG2 cells (ATCC) were maintained in D-MEM (Life Technologies) supplemented with FBS (JRS Scientific), Penicillin-Streptomycin and Glutamine (UCSF Cell Culture Facility). On the day of transfection, the cells were trypsinized, washed, and diluted to a density of 1 million cells/ml in Opti-MEM I Reduced Serum Media (Life Technologies). Ninety thousand cells and 125,000 cells were added to each well of a 96-well clear bottom black tissue culture plate (Greiner Bio-One) containing the transfection mixture for the promoter and enhancer assay respectively. The transfection mixture consisted of 50 ng of human PXR (*PXR* cDNA cloned into the pcDNA3.1(+) vector), 10 ng of pGL4.74, 21.5 ul Opti-MEM, 100 ng of reporter construct, and 0.48 ul of Lipofectamine LTX reagent (Life Technologies). After 18 hours of incubation at 37 degrees Celsius, the cells were washed with 150 ul Opti-MEM and the media was replaced with 100 ul of Opti-MEM supplemented with 10 uM Rifampin or 0.1% DMSO. After 24 hours of incubation, the cells were washed with PBS and the promoter assay cells were placed at −80 degrees Celsius for 30 minutes to lyse and the enhancer assayed cells were lysed with 25 ul of Passive Lysis Buffer (Promega). All LightSwitch promoter vectors were measured with the LightSwitch Luciferase Assay reagents (SwitchGear Genomics) following the manufacturers protocol. For enhancers, reporter activity was measured using the Dual-Luciferase Reporter Assay System (Promega). Both assays were measured on a microplate luminometer (Promega).

### Identification of common haplotypes from 1000 Genomes data

We used the 1000 Genomes March 2012 Interim release (), which contained genotypes for 379 White, 286 Asian, 181 Latino, and 246 African race/ethnicity individuals. For each gene RIR/GLP region, we identified haplotypes determined by computationally phasing the SNPs in the program PLINK v1.07 [75], again using the corresponding race/ethnicity.

### Accession numbers

ChIP-seq and RNA-seq data has been made publically available through NCBI (ChIP-seq BioProject ID: PRJNA239635; RNA-seq BioProject ID: PRJNA239637).

## Supporting Information

Figure S1 Distribution of PXR ChIP-seq peaks following rifampin treatment. (A) All genes. (B) Differentially expressed genes only.
(PDF)

Figure S2 Ingenuity Pathway Analysis of genes near rifampin induced regions (RIRs) shows genes involved in “PXR/RXR Activation”. Upregulated genes are colored in red and downregulated genes are colored in green as determined through either qPCR or RNA-seq. Fold differences are shown as a log2 ratio below each one of the differentially expressed genes. CYP2A6* represents the *CYP2A13* gene which is upregulated.
(PDF)

Figure S3 GLP1 and GLP2. (A)*CYP2C* locus showing both the RNA-seq and ChIP-seq results for DMSO (blue) and rifampin (red) treated hepatocytes. GLP1 and GLP2 are depicted by green lines above the *CYP2C* genes. (B) Common SNPs in GLP1 and GLP2.
(TIF)

Figure S4 *GSTA* locus showing both the RNA-seq and ChIP-seq results for DMSO (blue) and rifampin (red) treated hepatocytes. The cloned RIR46 fragment is depicted by a black rectangle upstream to *GSTA2*.
(TIF)

Figure S5 GLP5. (A)*UGT1A* locus showing both the RNA-seq and ChIP-seq results for DMSO (blue) and rifampin (red) treated hepatocytes. GLP5 is depicted by a green line above the *UGT1A* genes. (B) The location of common SNPs within GLP5.
(TIF)

Table S1 qPCR results for 84 genes known to be involved in drug response and RNA-seq results following vehicle or rifampin treatment.
(XLSX)

Table S2 ChIP-seq summary.
(XLSX)

Table S3 Promoter assay results.
(XLSX)

Table S4 GREAT analysis of rifampin induced regions (RIRs).
(XLSX)

Table S5 Ingenuity Pathway Analysis of genes near rifampin induced regions (RIRs).
(XLS)

Table S6 GWAS linked peaks (GLPs).
(XLSX)

Table S7 Enhancer assay results.
(XLSX)

Table S8 The various common haplotypes selected for differential enhancer assays, haplotype enhancer assays results and ANOVA analyses.
(XLSX)

Table S9 H3K4me1 and H3K27ac islands identified by alternate analysis in which the rifampin-treated sample was compared directly to the DMSO treatment as reference.
(XLSX)
