# Supplementary figures and images for: Genome-Wide Discovery of Drug-Dependent Human Liver Regulatory Elements
Source: PLoS Genet. 2014 Oct 2;10(10):e1004648. doi: 10.1371/journal.pgen.1004648 (PMC4183418; doi:10.1371/journal.pgen.1004648)

**A**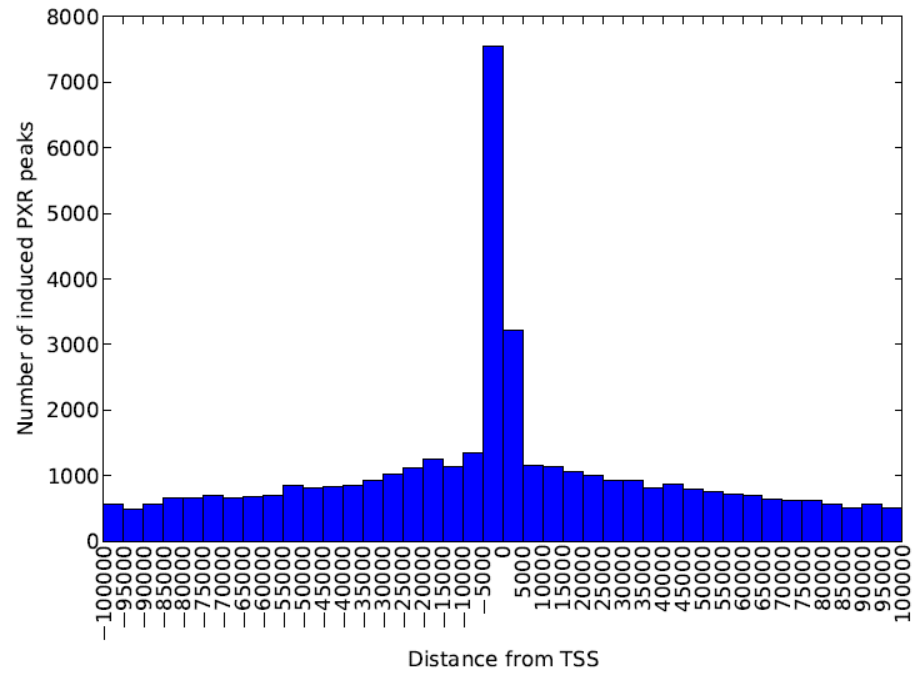**B**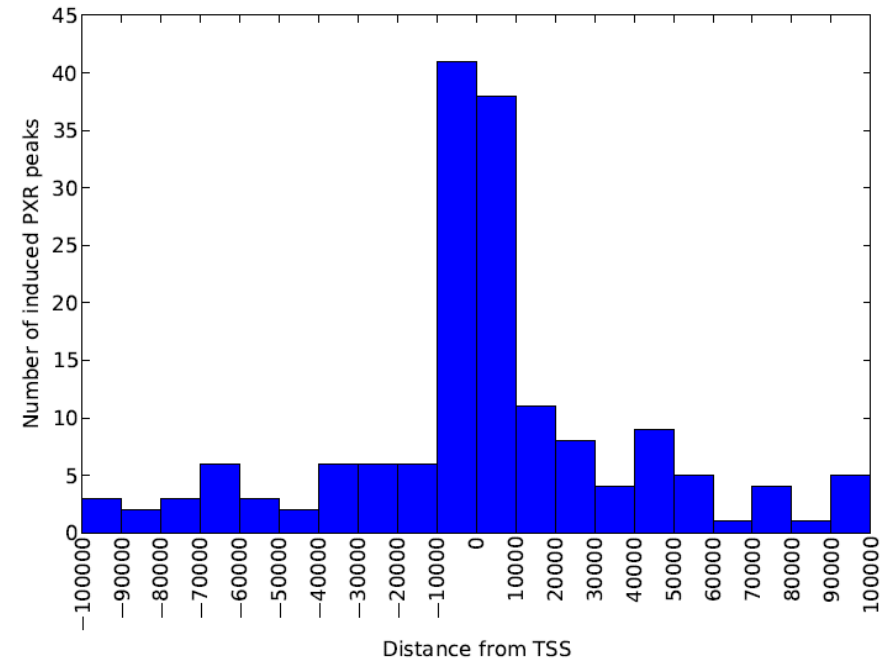

Supplement: Figure S1 — Distribution of PXR ChIP-seq peaks following rifampin treatment. (A) All genes. (B) Differentially expressed genes only. (PDF) [file pgen.1004648.s001.pdf]

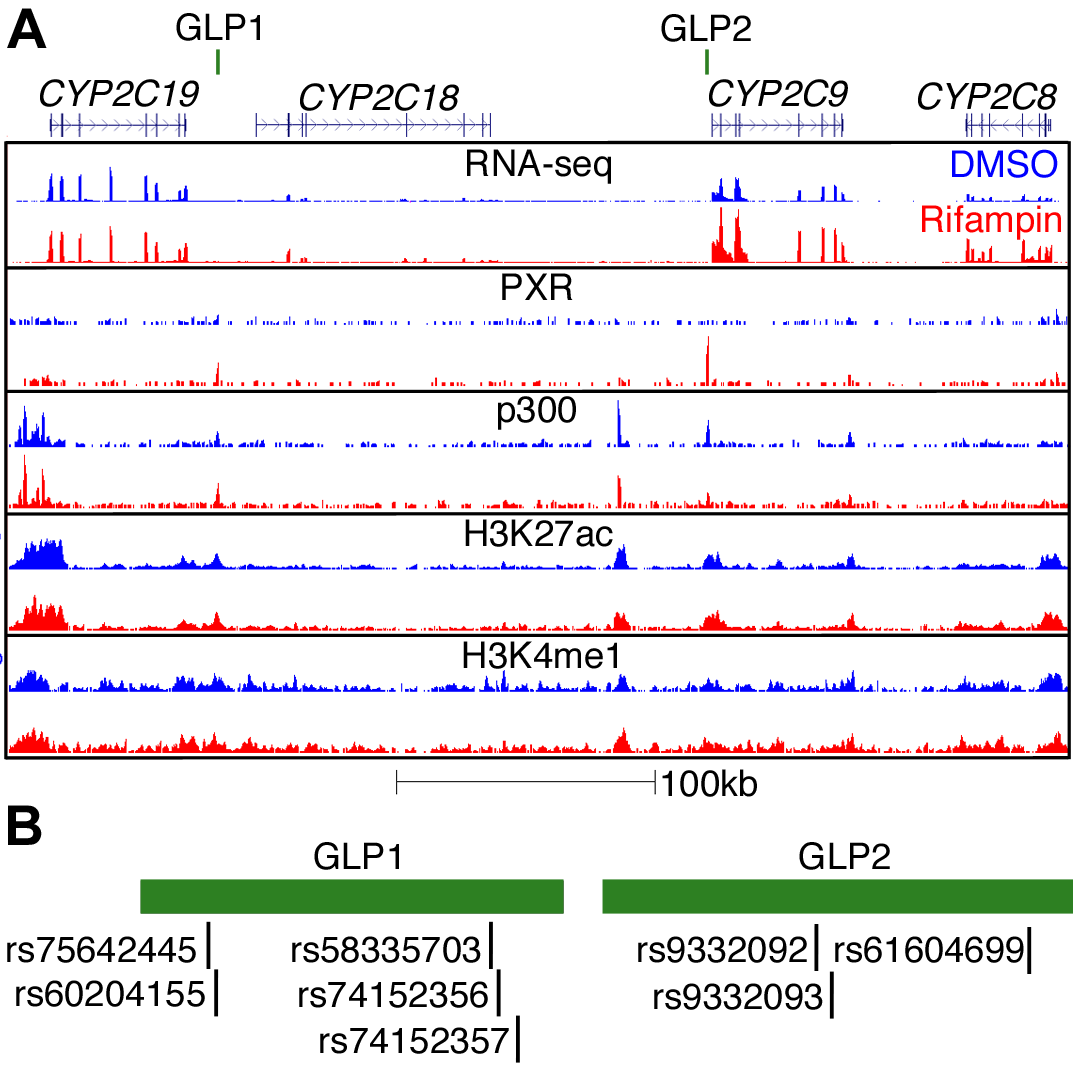

Supplement: Figure S3 — GLP1 and GLP2. (A)CYP2C locus showing both the RNA-seq and ChIP-seq results for DMSO (blue) and rifampin (red) treated hepatocytes. GLP1 and GLP2 are depicted by green lines above the CYP2C genes. (B) Common SNPs in GLP1 and GLP2. (TIF) [file pgen.1004648.s003.tif]

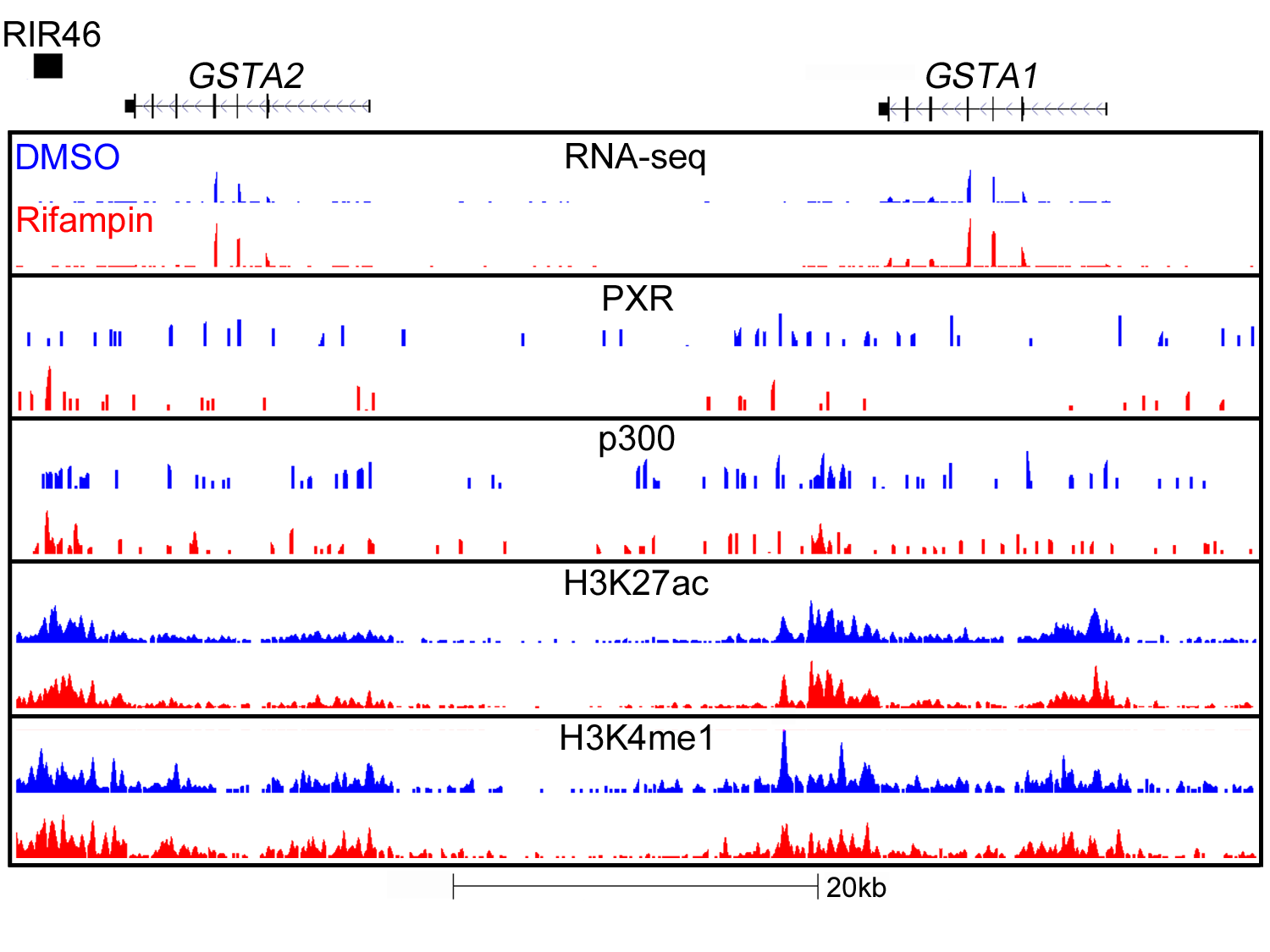

Supplement: Figure S4 — GSTA locus showing both the RNA-seq and ChIP-seq results for DMSO (blue) and rifampin (red) treated hepatocytes. The cloned RIR46 fragment is depicted by a black rectangle upstream to GSTA2. (TIF) [file pgen.1004648.s004.tif]

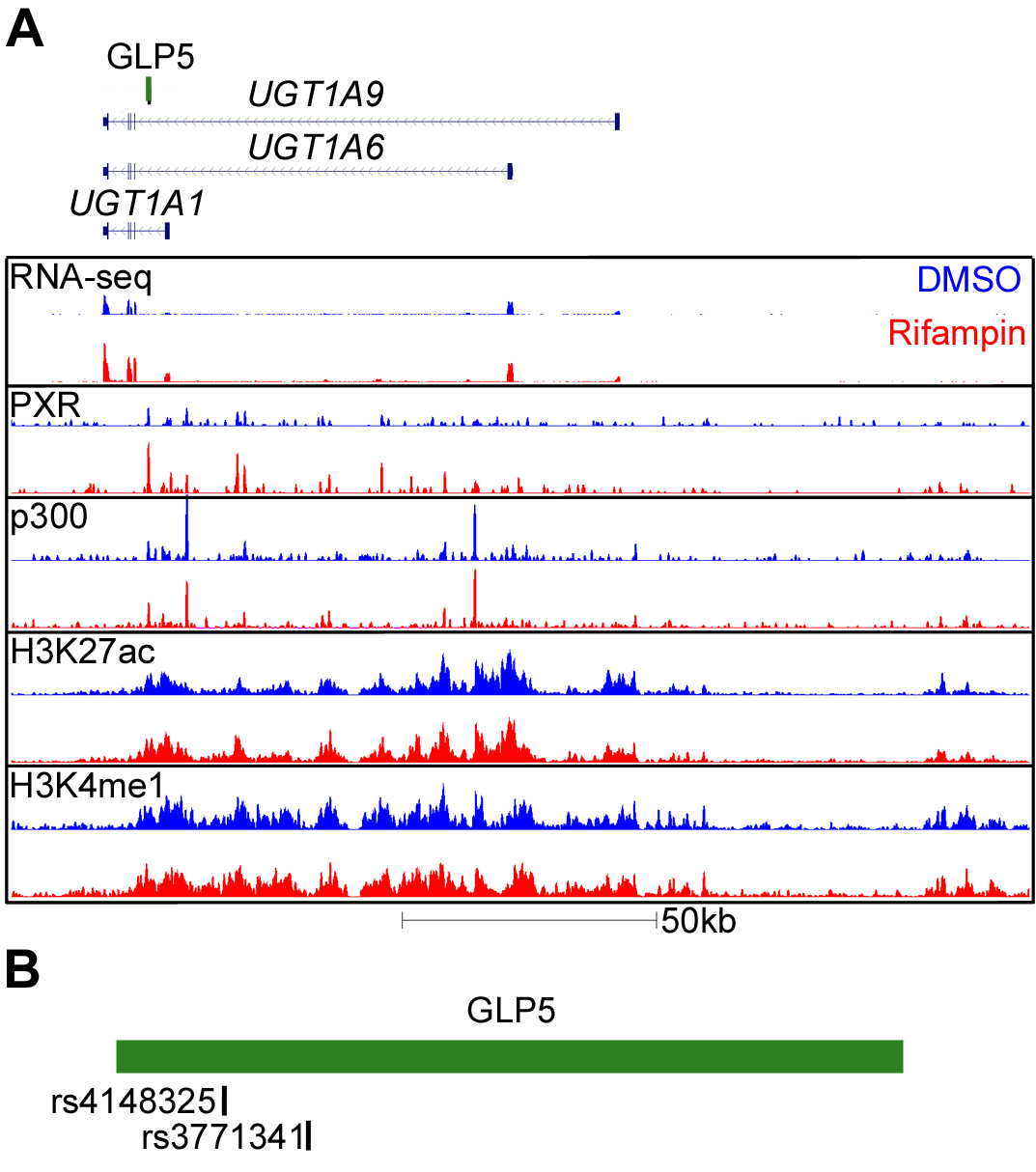

Supplement: Figure S5 — GLP5. (A)UGT1A locus showing both the RNA-seq and ChIP-seq results for DMSO (blue) and rifampin (red) treated hepatocytes. GLP5 is depicted by a green line above the UGT1A genes. (B) The location of common SNPs within GLP5. (TIF) [file pgen.1004648.s005.tif]
