# Supplementary material for: Genome-Wide Discovery of Drug-Dependent Human Liver Regulatory Elements
Source: PLoS Genet. 2014 Oct 2;10(10):e1004648. doi: 10.1371/journal.pgen.1004648 (PMC4183418; doi:10.1371/journal.pgen.1004648)
Supplement: Figure S2 — Ingenuity Pathway Analysis of genes near rifampin induced regions (RIRs) shows genes involved in “PXR/RXR Activation”. Upregulated genes are colored in red and downregulated genes are colored in green as determined through either qPCR or RNA-seq. Fold differences are shown as a log2 ratio below each one of the differentially expressed genes. CYP2A6* represents the CYP2A13 gene which is upregulated. (PDF) [file pgen.1004648.s002.pdf]

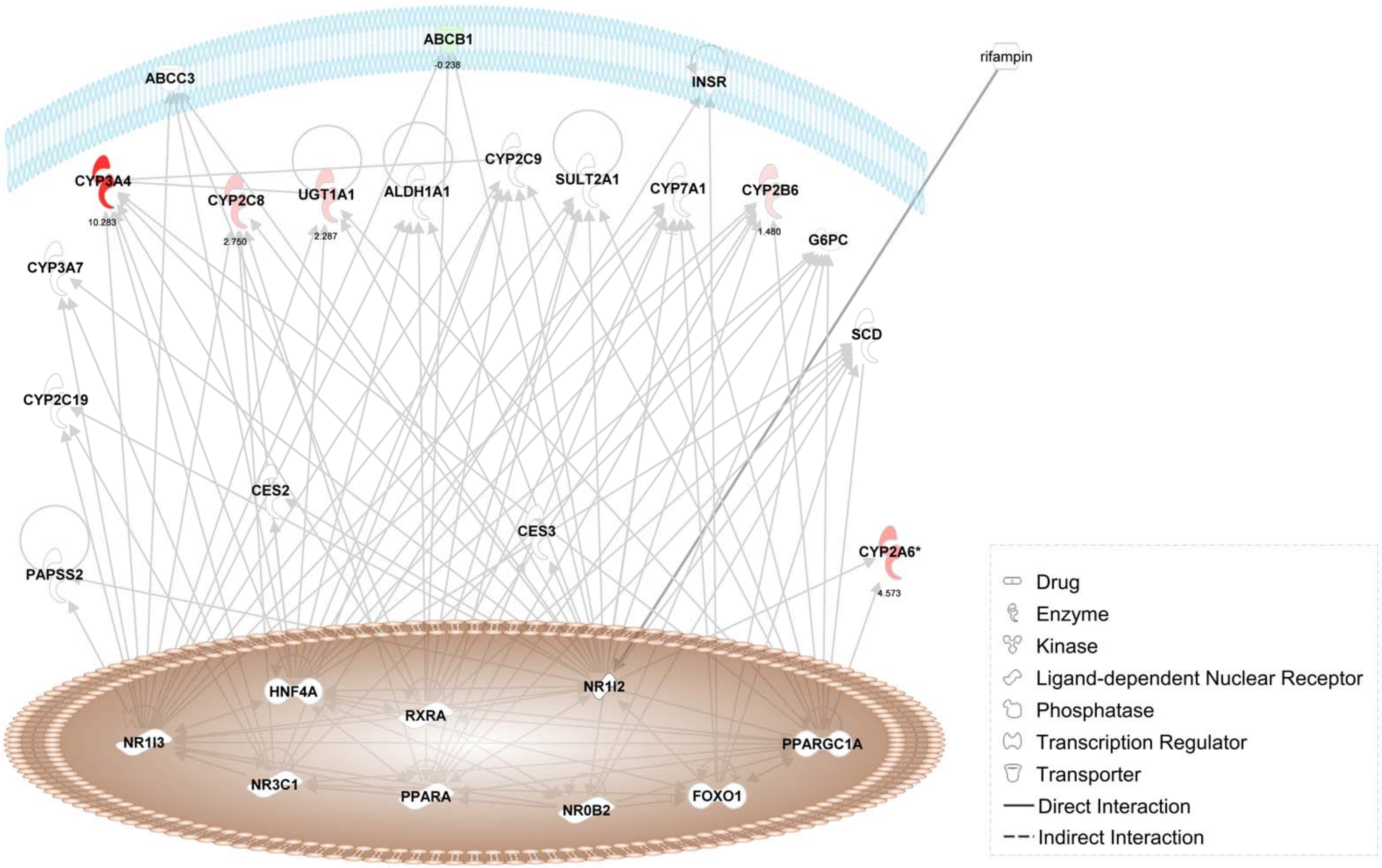

ABCB1
-0.238
rifampin
ABCC3
INSR
CYP2C9
SULT2A1
CYP3A4
10.283
CYP2C8
2.750
UGT1A1
2.287
ALDH1A1
CYP7A1
CYP2B6
1.480
G6PC
CYP3A7
SCD
CYP2C19
CES2
CES3
CYP2A6*
4.573
PAPSS2
HNF4A
NR1I2
RXRA
NR1I3
PPARGC1A
NR3C1
PPARA
NR0B2
FOXO1
Drug
Enzyme
Kinase
Ligand-dependent Nuclear Receptor
Phosphatase
Transcription Regulator
Transporter
Direct Interaction
Indirect Interaction
